# Synthesis of Chiral Gold Nanostructures on Gold‐Coral‐Coated Transparent Conductive Oxide Substrates via [AuCl_4_]^–^ and Cysteine Interactions for Enhanced Chiral Sensing

**DOI:** 10.1002/smll.75471

**Published:** 2026-09-03

**Authors:** Kenta Nakagawa, Junhe Wang, Tianji Zhang, Yuta Shiga, Olga Guselnikova, Takuya Nakanishi, Yusuke Yamauchi, Toru Asahi

**Affiliations:** ^1^ Comprehensive Research Organization Waseda University Tokyo Japan; ^2^ Faculty of Science and Engineering Waseda University Tokyo Japan; ^3^ International Center for Materials Nanoarchitectonics (WPI‐MANA) National Institute for Materials Science (NIMS) Tsukuba Ibaraki Japan; ^4^ Department of Materials Science and Engineering School of Engineering Nagoya University Nagoya Japan; ^5^ Australian Institute for Bioengineering and Nanotechnology (AIBN) The University of Queensland (UQ) Brisbane QLD Australia; ^6^ Research Organization for Nano & Life Innovation Waseda University Tokyo Japan

**Keywords:** Au corals, chiral sensing, SERS

## Abstract

Chirality appears in many molecules, crystals, and nanostructures and plays an important role in their physicochemical and biological functions. Because biological systems exhibit a chiral bias, they respond differently to each chiral enantiomer. Therefore, it is very important to develop methods or devices that can discriminate the enantiomers easily. Metallic nanostructures such as nanoparticles, nanorods, and mesoporous films exhibit significantly enhanced optical responses due to localized surface plasmon resonance. The optical properties of these nanostructures are highly dependent on their size, shape, and elemental composition, all of which can be precisely controlled through optimized chemical synthesis. In this study, we developed a hierarchical nanostructure of chiral Au nanostructures on Au coral structures, which were synthesized on transparent conductive oxide substrates using only electrochemical techniques. By considering both the substitution of chloride ions in [AuCl_4_]^−^ with cysteine in the electrolyte solution and the subsequent reduction of the Au(III) complexes, we successfully controlled the morphology of the Au nanostructures, which exhibit circular dichroism. Furthermore, we explored the potential for chiral discrimination using these substrates. The hierarchical chiral Au nanostructures on Au coral structures enhanced the SERS intensity relative to Au corals alone and produced enantiomer‐dependent SERS responses for L‐ and D‐phenylalanine, demonstrating their potential for chiral discrimination.

## Graphical abstract

We developed hierarchical chiral Au nanostructures on Au coral structures on transparent conductive oxide substrates using only electrochemical methods. By controlling the interaction between [AuCl_4_]^–^ and cysteine, we successfully tuned the morphology and circular dichroism of the resulting Au nanostructures. These substrates enhanced relative SERS intensity and yielded enantiomer‐dependent SERS responses for phenylalanine, demonstrating their potential for chiral discrimination.

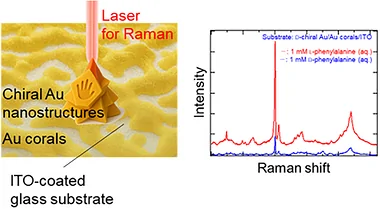

## 1 Introduction

“Chirality” is a property of a three‐dimensional object or phenomenon that cannot be superimposed on its mirror image by any combination of translations and proper rotations [1, 2]. Chirality appears in many molecules, crystals, and nanostructures and plays important roles not only in determining the structures of chemical substances but also in their biological functions. Since biological systems exhibit a chiral bias (e.g., biological systems predominantly use l‐amino acids and d‐sugars), they respond differently to each chiral isomer (enantiomer) [1, 2]. In the case of chiral pharmaceuticals such as thalidomide, the enantiomers can exhibit different pharmacological activities and toxicities [3, 4]. Therefore, it is very important to develop methods or devices that can discriminate the enantiomers easily.

Because chiral molecules exhibit a polarization‐dependent interaction with light, optical analytical techniques such as circular dichroism (CD) spectroscopy and polarimetry are well suited for sensing and characterizing chirality [5, 6]. A CD spectrophotometer measures the difference in absorption between left‐ and right‐circularly polarized light, while a polarimeter measures the rotation of the plane of incident linearly polarized light, which arises from the difference in refractive index between left‐ and right‐circularly polarized light. Since circularly polarized light is itself chiral, these differences in absorption and/or refractive index originate from the interaction between chiral circularly polarized light and chiral molecules. However, this interaction is intrinsically weak because the size of molecules we focus on is approximately 10^3^–10^5^ times smaller than the wavelength of the circularly polarized light, resulting in very small signals on the order of millidegrees [7, 8]. Consequently, large sample quantities are required to achieve an adequate signal‐to‐noise ratio. In contrast, metallic nanostructures such as nanoparticles, nanorods, and mesoporous structures exhibit significantly enhanced optical responses due to localized surface plasmon resonance, which is the coherent oscillation of free electrons in the metal induced by an oscillating electromagnetic field. The optical properties of these nanostructures are highly dependent on their size, shape, and chemical composition, all of which can be precisely controlled through optimized chemical synthesis. Therefore, the controlled fabrication of nanostructures is an important issue in materials chemistry for achieving unique and enhanced optical responses.

Numerous chemical synthesis methods have been developed for fabricating metal nanoparticles and nanorods [9, 10]. Certain chiral molecules, such as amino acids and peptides exhibit different binding affinities to specific crystal facets of Au, which can be utilized as structure‐directing agents to control the morphology of the nanostructures. However, precise control of the shape and chirality of Au nanostructures through chemical synthesis remains challenging. This is because a wide range of parameters must be optimized, including (1) the concentration of the Au precursor, (2) the type and concentration of the surfactant or stabilizer, (3) the type and concentration of the reducing agent, (4) the type and concentration of the chiral additive, and (5) reaction conditions such as temperature, pH, reaction time, and timing of reagent addition. Achieving highly controlled and well‐defined chiral nanostructures requires extensive experimental efforts and careful optimization of these variables. For the various potential applications of chiral nanostructures mentioned above, it is often desirable to immobilize them onto solid substrates.

Compared to chemical synthesis, electrochemical techniques offer several advantages for synthesizing Au films. These include (1) the ability to synthesize films simply by applying a potential to the working electrode (so‐called electrodeposition), (2) precise control of film thickness through deposition time, and (3) the facile formation of hierarchical nanostructures through sequential electrodeposition steps. Notably, Yamauchi et al. developed an electrochemical method for synthesizing mesoporous nanostructures using an electrolyte solution containing block‐copolymer micelles as soft templates [11, 12, 13]. Owing to their three‐dimensional metallic framework with numerous nanoscale tips, these mesoporous nanostructures demonstrated significant potential for surface‐enhanced Raman spectroscopy (SERS) in molecular detection [14, 15]. This enhancement is attributed to the presence of multiple plasmonic hot spots within the mesopores and along the narrow walls separating them. Furthermore, Gu et al. developed an electrochemical method for synthesizing chiral plasmonic Au nanostructures using an electrolyte solution containing the amino acid cysteine as a chiral additive [16]. While they successfully synthesized chiral Au nanostructures on an indium tin oxide (ITO)‐coated glass substrate, the synthesis mechanism was not elucidated in detail, which may raise concerns about the reproducibility of the method. In addition, they demonstrated enantioselective sensing by observing shifts in CD spectra, which arise from dipole interactions between the chiral analyte molecules and the chiral Au nanostructures. However, unlike SERS, this method does not directly provide detailed information about the specific vibrational modes of the analyte molecules, which is essential for molecular identification.

In this study, we developed a hierarchical nanostructure of chiral Au nanostructures on Au corals, which were synthesized on an ITO‐coated glass substrate using only electrochemical techniques. Here, Au corals are the structures in the initial stages of mesoporous Au film deposition. By considering both the substitution of chloride ions in [AuCl_4_]^−^ with cysteine and the subsequent reduction of the Au(III) complexes, we successfully controlled the morphology of the Au nanostructures, which exhibit CD. Furthermore, by measuring the SERS spectra of amino acid analytes on the chiral Au nanostructures on Au corals, we explored the potential and detection mechanism for chiral molecule analysis by using the substrates.

## 2 Methods

### 2.1 Synthesis of Au Corals on ITO‐Coated Glass Substrates

To prepare the electrolyte solution for Au coral deposition, 10 mg of polystyrene‐*b*‐poly(ethylene oxide) (PS(18,000)‐*b*‐PEO(7,500)) was dissolved in 3 mL of tetrahydrofuran (THF) at 40°C [11, 12, 13]. Subsequently, 1.5 mL of ethanol, 1 mL of 40 mM HAuCl_4_ (aq.), and 2.5 mL of deionized water (DIW) were added dropwise to the PS‐*b*‐PEO solution under gentle stirring to form spherical micelles of the block copolymer. The resulting transparent bright‐yellow electrolyte solution was used for electrodeposition on an ITO‐coated glass substrate. Electrochemical deposition was performed using a conventional electrochemical potentiostat (ALS1040C, BAS) in a three‐electrode configuration, consisting of a platinum wire as the counter electrode, an Ag/AgCl reference electrode, and the ITO‐coated glass substrate as the working electrode. Au electrodeposition was carried out at a constant potential of −0.5 V (vs. Ag/AgCl) for 200 s at room temperature. After deposition, the micelles acting as soft templates were removed by washing with THF at 40°C followed by rinsing with DIW.

### 2.2 Synthesis of Chiral Au Nanostructures on Au Corals

Chiral Au nanostructures were synthesized on the Au corals prepared in advance using an electrolyte solution containing 0.1 m KCl (aq.), 2 mm HAuCl_4_ (aq.), and 0.4 mm L or D‐cysteine (aq.) [16]. Electrochemical deposition was conducted under the same three‐electrode configuration. The deposition was performed at a constant potential of 0 V (vs. Ag/AgCl) for 500 s at room temperature. After the deposition, the chiral additive (cysteine) was removed by rinsing with DIW.

### 2.3 Characterization

The morphologies of the synthesized Au nanostructures were observed using scanning electron microscopy (SEM) (SU‐8240, Hitachi High‐Tech) at an accelerating voltage of 15 or 20 kV. CD spectra were measured using a CD spectrophotometer (J‐820, JASCO) to evaluate the chiral structures of both the electrolyte solutions and the synthesized nanostructures. The crystal structures were characterized by X‐ray diffraction (XRD) (RINT‐UltimaIII, Rigaku) with Cu K*_α_* radiation (*λ* = 1.5418 Å). For the ^13^C NMR measurements, a highly concentrated aqueous solution containing 500 mm KCl, 20 mm HAuCl_4_, and 38 mm L‐cysteine was used to obtain sufficient signal intensity, given the low natural isotopic abundance of ^13^C (∼1.1%) [17]. For ^1^H NMR spectroscopy, solutions were prepared in D_2_O containing 0.1 m KCl, 2 mm HAuCl_4_, and 0.4 mm of either L‐ or D‐cysteine. Liquid‐state ^13^C NMR and ^1^H NMR spectra were recorded on a ADVANCE 600 NEO (Bruker) spectrometer at resonance frequencies of 150.9 and 600.1 MHz, respectively. Au *L*_3_‐edge XAFS measurements were carried out at beamline BL11S2 of the Aichi Synchrotron Radiation Center (Aichi, Japan) [18]. The measurements were conducted in transmission mode using a Si(111) double‐crystal monochromator. The solution sample was loaded in the PMMA cell and mounted between the *I*_0_ and *I*_1_ ionization chambers. The XANES and EXAFS data were processed using the Athena software program, a part of the Demeter package, which provides an interface to the IFEFFIT library [19]. All spectra were normalized such that the edge step, defined as the difference between the average pre‐edge and post‐edge regions, was set to 1.0. The EXAFS oscillations were isolated by subtracting a spline fit to the post‐edge background. Fourier transforms were carried out over the k‐range of 3–10 Å^−1^ using a Hanning window.

### 2.4 SERS Spectra Measurements

Raman spectra were measured with a Raman microscope (inVia RefreX, Renishaw) using 785 nm laser excitation and an accumulation time of 30 s. Raman excitation and signal collection were performed using a 50× objective lens (*NA*: 0.5), yielding an spot diameter *d* of *d* ∼ *λ*/2*NA* ∼ 1 µm. In this study, the substrates were immersed in the analyte solution, and measurements were performed in situ.

To investigate the chiral discrimination capability of the films, the measurements for l‐ and d‐phenylalanine were carried out on both types of chiral Au nanostructures synthesized on the Au corals using l‐ and d‐cysteine, respectively, under identical experimental conditions. The same batches of l‐ and d‐phenylalanine solutions were used for the measurements. Thus, four combinations, including l‐chiral Au/coral Au/ITO substrate for l‐Phe, and l‐chiral Au/coral Au/ITO substrate for d‐Phe, d‐chiral Au/coral Au/ITO substrate for L‐Phe, and d‐chiral Au/coral Au/ITO substrate for D‐Phe, were evaluated to compare the interaction between the chiral Au/coral Au/ITO substrates and the two phenylalanine enantiomers.

## 3 Results and Discussion

Au corals were synthesized on ITO‐coated glass substrates using a method similar to that developed by Li et al. for the synthesis of mesoporous Au films. The electrochemical deposition technique provides the advantage of facile and precise control of the growth rate, enabling fine‐tuning of the film thickness by simply adjusting the deposition time. According to previous studies [11, 13], a film thickness of approximately 70 nm was obtained after 600 s of deposition at a constant potential of −0.5 V (vs. Ag/AgCl). Considering the inherent UV–vis light absorption characteristics of Au, a shorter deposition time of 200 s was selected to fabricate thinner Au corals. This shortened deposition time was intended to decrease the thickness of the first Au layer (<50 nm) and to minimize the overall light absorption. The results of two consecutive linear sweep voltammetry (LSV) measurements conducted before the electrodeposition and the chronoamperometry measurement conducted during the electrodeposition are shown in Figure S1A. The SEM image of the Au corals synthesized on the ITO‐coated glass substrate is shown in Figure 1B.

**FIGURE 1:**
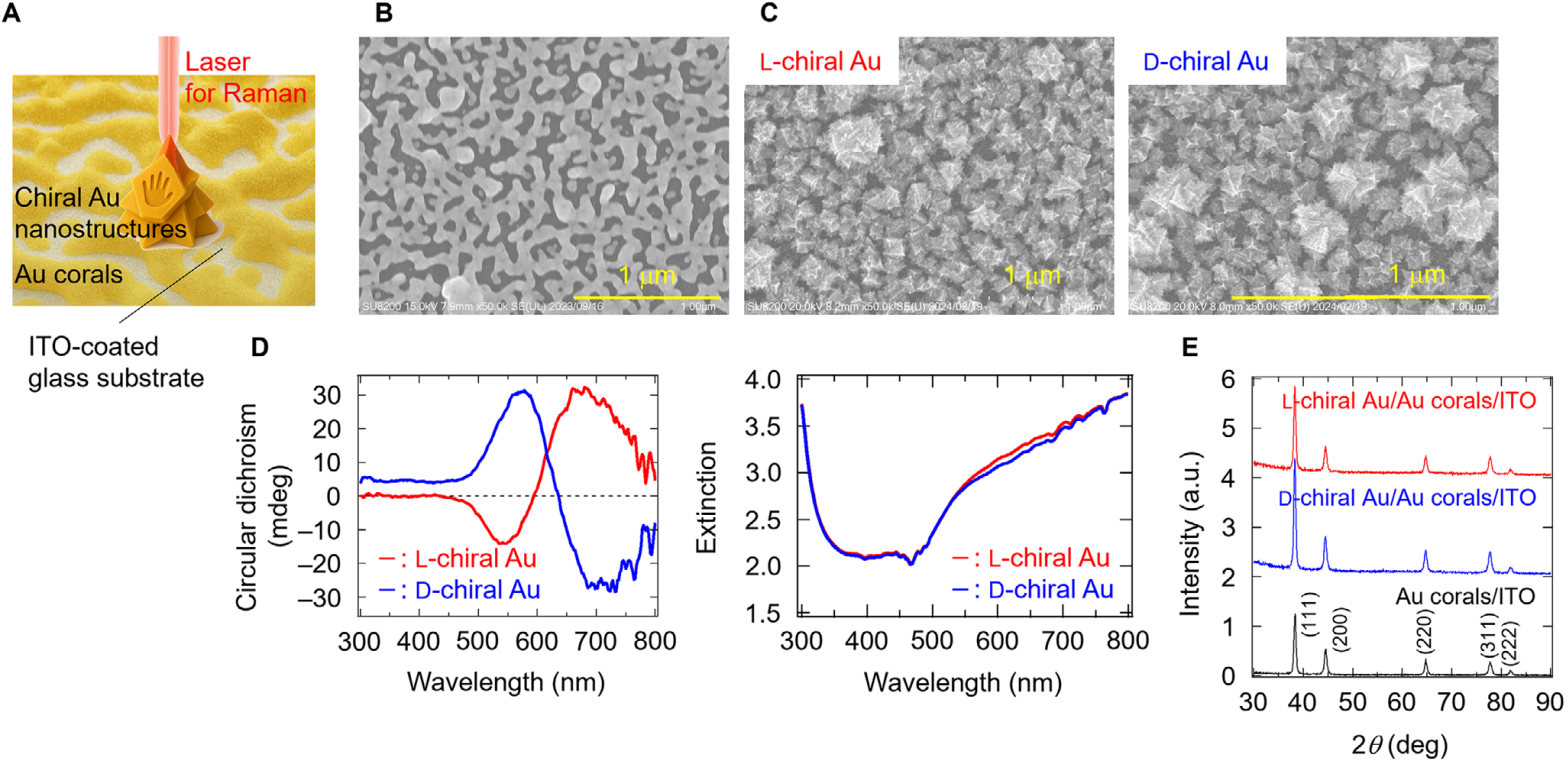
(A) Schematic image of the chiral Au nanostructures on Au corals/ITO‐coated glass substrates. (B,C) SEM images of Au corals (B), and Au nanostructures synthesized with L‐cysteine (C, left) and D‐cysteine (C, right). (D) CD and UV–vis spectra of the chiral Au nanostructures synthesized on Au corals. (E) XRD patterns of Au corals (black) and chiral Au nanostructures synthesized on Au corals (red: L‐chiral Au, blue: D‐chiral Au). Here, chiral gold nanostructures synthesized using L‐ and D‐cysteine on the Au corals are referred to as “L‐chiral Au” and “D‐chiral Au”, respectively.

Chiral Au nanostructures were synthesized on the Au corals in a second electrodeposition step. The electrolyte composition followed the method reported by Gu et al. for the synthesis of chiral Au nanostructures [16]. Interestingly, we unexpectedly found that the chiral Au nanostructures exhibiting strong CD were reproducibly obtained when the electrodeposition was initiated 20 min after mixing solutions of HAuCl_4_ (aq.) and L or D‐cysteine (aq.) at room temperature. The synthesis mechanism can be discussed in terms of chloride ion substitution of the [AuCl_4_]^−^ complex by cysteine, followed by the reduction of the resulting Au(III) complexes. The results of two consecutive linear sweep voltammetry (LSV) measurements conducted before the electrodeposition and the chronoamperometry measurement conducted during the electrodeposition are shown in Figure S1B,C. The SEM image of the chiral Au nanostructures synthesized on Au corals and the corresponding CD spectra are shown in Figure 1C,D, respectively. The films exhibited negative (positive) CD signals around 550 nm and positive (negative) signals around 650 to 700 nm, depending on the chirality of cysteine used during synthesis. It should be noted that cysteine molecules were removed from the surface by rinsing with DIW. Since cysteine does not exhibit any absorption above 300 nm, the observed CD signals are attributed to plasmonic resonance. Although the Au nanostructures exhibit helical or spiral‐like morphologies, it remains challenging to clearly distinguish the handedness of each individual nanostructure based solely on their appearance. However, the formation of chiral structures is likely associated with the emergence of intrinsically chiral high Miller index surfaces ({*hkl*}, where *h* ≠ *k* ≠ *l* ≠ 0), which can arise from the presence of kink sites during nanoparticle growth [20, 21]. Gu et al. previously reported that electrodeposition under stirring conditions (300 rpm) enhanced the CD intensity compared to deposition without stirring [16]. We also confirmed that samples synthesized with stirring exhibited large CD signals (Figure S2).

To confirm that the observed circular dichroism signals and chiral discrimination capabilities originate from the intrinsic properties of the gold nanostructures, rather than from interference due to residual organic species or gold oxides, we performed XPS measurements for the substrates and compared the obtained spectra with those of an Au substrate modified with a self‐assembled monolayer (SAM) of cysteine, which is representative of surfaces containing adsorbed cysteine molecules (Figure S3). The S 2*p* and N 1*s* peaks were substantially weaker than those observed for the cysteine SAM‐modified substrate measured under identical conditions, indicating that the influence of residual cysteine was negligible. In addition, no evidence of gold oxide formation was observed in the Au 4*f* spectrum. The peaks at 84.0 and 87.7 eV were assigned to the Au 4*f*_7/2_ and 4*f*_5/2_ components, respectively, and there was neither tailing at higher binding energies nor any additional peaks that indicate the presence of gold oxide, confirming that Au was present in the metallic state (Au(0)) [22]. Thus, we ruled out significant interference from residual cysteine or gold oxides.

To characterize the crystallographic orientation of the electrodeposited Au nanostructures, x‐ray diffraction (XRD) measurements were performed to estimate the preferred orientation and relative abundance of different crystal facets. Figure 1E shows the XRD patterns of three samples: Au corals synthesized on ITO‐coated glass substrates, and chiral Au nanostructures synthesized on Au corals (red: L‐chiral Au, blue: D‐chiral Au). For each sample, diffraction peaks corresponding to (111), (200), (220), (311), and (222) planes were observed within the measured 2*θ* range. The figure reveals that the intensity ratio of the (200) peak to the (111) was 0.488 for the Au corals on ITO‐coated glass substrates, whereas it decreased to 0.342 and 0.397 for the chiral Au nanostructures on Au corals. This reduction in the (200)/(111) intensity ratio suggests that cysteine may selectively adsorb onto the (111) surfaces, passivating them and thereby suppressing their growth, which results in the preferential exposure of (111) facets in Au nanostructures due to kinetically controlled growth. In the absence of cysteine, no specific adsorbates are present to selectively stabilize particular Au surfaces, allowing the Au nanostructures to grow in multiple orientations. This results in the formation of larger, more spherical Au domains. As shown in Figure S4, the intensity ratios of the (200) to (111) peaks for chiral Au nanostructures synthesized on ITO‐coated glass substrates were 0.358 and 0.307, respectively, exhibiting the same trend as those on Au corals/ITO‐coated glass substrates.

As mentioned above, we unexpectedly found that the chiral Au nanostructures exhibiting strong CD were reproducibly obtained when the electrodeposition was initiated 20 min after mixing solutions of HAuCl_4_ (aq.) and L or D‐cysteine (aq.) at room temperature as shown in Figure 2A. Notably, the CD intensity increased up to 20 min, but decreased to nearly zero after 40 min. To understand this phenomenon, we attempted to elucidate the underlying mechanism from the perspective of the interaction between [AuCl_4_]^−^ complex and cysteine. The chemistry and stability of the gold complexes such as [AuCl_4_]^−^ and [Au(CN)_4_]^−^ have been extensively studied [23, 24, 25, 26, 27, 28, 29, 30]. In such gold complexes, central Au(III) acts as a soft Lewis acid that prefers interactions with soft ligands [23]. It is also well established that Au(III) can be reduced to Au(I) by softer nucleophiles [23]. Reactions of [AuCl_4_]^−^ or [Au(CN)_4_]^−^ with cysteine showed that ligand substitution from Cl^−^ to cysteine generally proceeds more rapidly than reduction [23, 24]. The stepwise Au(III) reduction mechanism including the ligand substitution between gold complexes and cysteine has been studied [23, 24, 25, 26, 27, 28, 29] but little attention has been paid to the structural aspects of this interaction from the viewpoint of chirality. Therefore, we measured the time dependence of the CD spectra of the electrolyte aqueous solution containing KCl, HAuCl_4_ and L or D‐cysteine.

**FIGURE 2:**
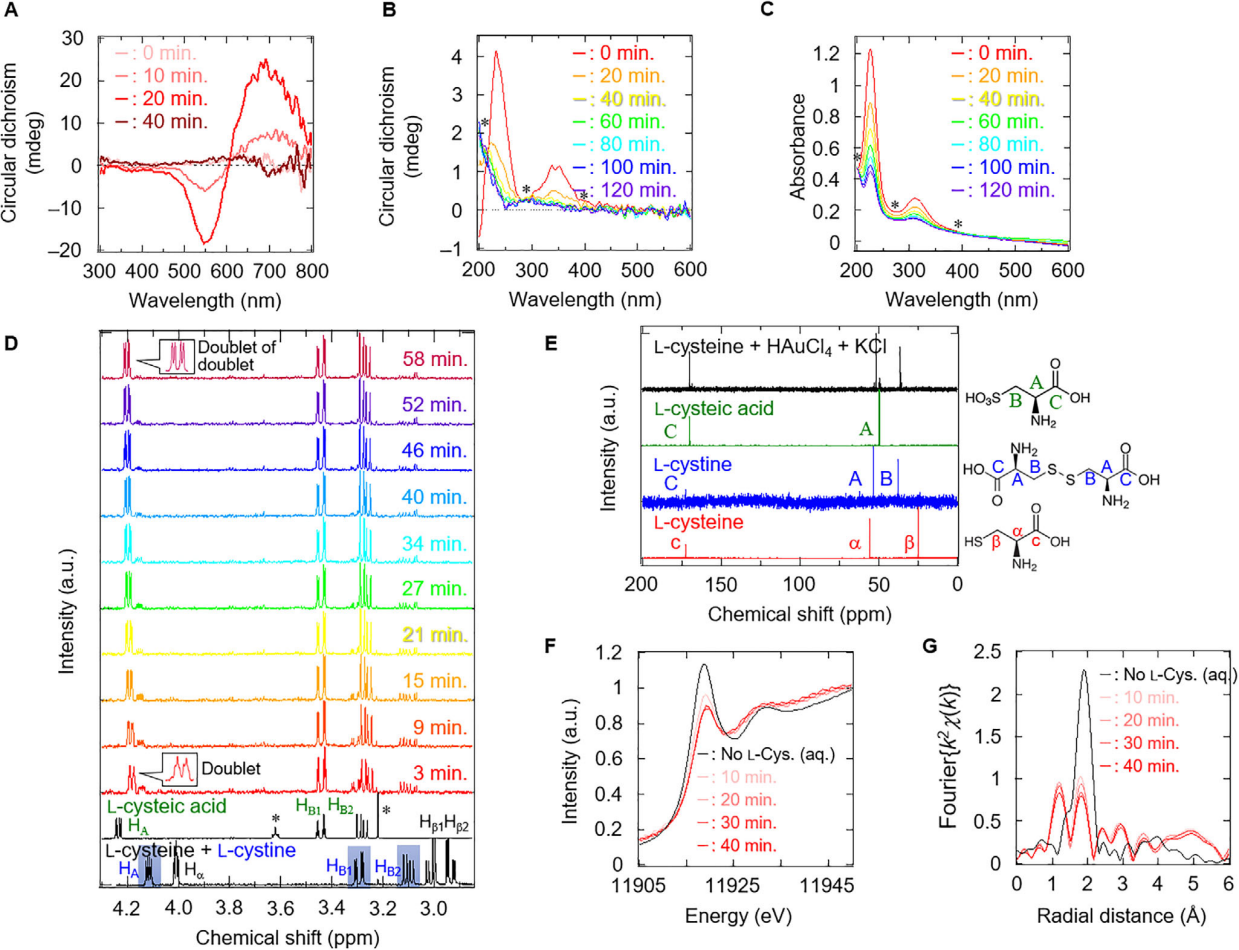
Time‐dependent spectroscopic and structural analyses of the interaction between [AuCl_4_]^−^ and cysteine. (A) CD spectra of chiral Au nanostructures synthesized on Au corals as a function of the waiting time between the mixing of aqueous KCl, HAuCl_4_, and L‐cysteine solutions and the start of electrodeposition. (B,C) Time‐dependent CD (B) and UV–vis (C) spectra of the mixed aqueous solution containing 15 mm KCl, 0.6 mm HAuCl_4_, and 0.3 mm L‐cysteine at room temperature. The asterisks indicate the isosbestic point. (D) Time‐dependent ^1^H NMR spectra of the mixed D_2_O solution containing 0.1 m KCl, 2 mm HAuCl_4_, and 0.4 mm L‐cysteine. (E) ^13^C NMR spectrum of the mixed aqueous solution containing 500 mm KCl, 20 mm HAuCl_4_, and 38 mm L‐cysteine. (F,G) XANES (F) and Fourier‐transformed EXAFS (G) spectra before and after mixing 2 mm HAuCl_4_ and 0.4 mm L‐cysteine.

We prepared an aqueous solution containing 15 mm KCl, 0.6 mm HAuCl_4_, and 0.3 mm of either L‐ or D‐cysteine for CD and UV–vis measurements. Here, the concentration of the solution used for the CD and UV–vis measurements differs from that of the electrolyte solution. These concentrations were selected to resolve signals from both cysteine and Au species. The CD and UV–vis spectra of HAuCl_4_ (aq.) and L‐cysteine (aq.), respectively are shown in Figure S5. A CD peak corresponding to the *n*‐π* transition of the cysteine molecule is observed around 200 nm, while absorption peaks correspond to the charge transfer transitions (*p*‐*d* transitions) were observed around 222 and 294 nm [31]. Immediately after mixing of HAuCl_4_ (aq.) and cysteine (aq.), ligand substitution from [AuCl_4_]^−^ to [cysteine‐AuCl_3_]^−^ and [(cysteine)_2_‐AuCl_2_]^−^ complexes likely occurred, as evidenced by dramatic changes in the shapes of CD and UV–vis spectra as shown in Figure 2B,C (Figure S6 for D‐cysteine). CD and absorption peaks observed at 232 and 226 nm, and 338 and 310 nm, are probably attributed to the ligand‐to‐metal charge transfer (LMCT) transition related to the Au‐S bond complex formation in [cysteine‐AuCl_3_]^−^ and [(cysteine)_2_‐AuCl_2_]^−^, respectively [24]. Subsequently, the intensities of these CD and absorption peaks gradually decreased. Here, isosbestic points were observed in both the CD and absorption spectra, as indicated by asterisks. The presence of an isosbestic point generally suggests that the observed spectral changes are dominated by the interconversion of two spectroscopically distinguishable species, although the presence of additional intermediates or minor species cannot be excluded. This indicates that the time‐dependent spectral changes can be predominantly described by a reaction involving two major chemical species before and after the reaction. Therefore, these spectral changes suggest that a reduction process from Au(III) to Au(I) is involved in the reaction system, leading to the formation of Au(I)–thiolate species, which may coexist in equilibrium with chloride‐coordinated Au(I) species such as [Au(I)Cl_2_]^−^ derived from [cysteine–AuCl_3_]^−^ and [(cysteine)_2_–AuCl_2_]^−^ complexes. After 40 min, the shape of the CD spectra was completely altered, and a new peak appeared at 200 nm. Based on previous reports and our NMR measurements, the following reaction mechanism is proposed. The reaction is initiated by the substitution of chloride ligands in [AuCl_4_]^−^ by cysteine, as indicated by the CD and absorption peaks observed immediately after mixing. Simultaneously, oxidative formation of the cysteine disulfide, i.e., cystine formation, may occur through an equilibrium reaction involving two adjacent [cysteine‐AuCl_3_]^−^ species. Subsequently, disproportionation reactions [23, 24, 27, 28, 29, 30] may occur, generating [Au(III)Cl_4_]^−^ and [Au(I)Cl_2_]^−^, while oxidized cysteine products, including cysteine sulfenic acid (Cys‐SOH), cysteine sulfinic acid (Cys‐SO_2_H) and cysteine sulfonic acid (cysteic acid, Cys‐SO_3_H), may be gradually generated [29, 30]. Over time, the concentration of cystine decreased, whereas the amount of oxidized cysteine products gradually increased. After 40 min, cysteine was consumed, and the CD peak attributed to cysteic acid was observed. Following cysteine consumption, cysteine sulfenic acid and cysteine sulfinic acid may have continued to mediate the reduction of [Au(III)Cl_4_]^−^ to [Au(I)Cl_2_]^−^, accompanied by a gradual decrease in the UV–vis absorption peaks. Similar Au(I)‐containing structures have been reported in supramolecular assemblies and intermediates during the synthesis of gold‐thiolate coordinated oligomers and nanoclusters [32]. According to previous reports, the strong CD signals cannot be explained by simple coupling between cysteine and the LMCT bands. Instead, they are likely attributed to the synergistic interplay between electrostatic interactions among the –SR side chains and the aurophilic attraction between Au(I) atoms within the Au(I)‐cysteine polymeric backbone, which may result in the formation of helical structures.

In order to analyze the interaction between [AuCl_4_]^−^ and cysteine, with a particular focus on the structural changes of the cysteine in the solution, ^1^H and ^13^C nuclear magnetic resonance (NMR) spectra were measured. For the ^1^H NMR measurements, solutions were prepared in D_2_O containing 0.1 m KCl, 2 mm HAuCl_4_, and 0.4 mm of L‐cysteine. Here, the concentrations of KCl, HAuCl_4_, L‐cysteine used for the ^1^H NMR measurements were identical to those used in the electrolyte solution. The time dependence of the ^1^H NMR spectra are shown in Figure 2D. According to the ^1^H NMR spectra of cysteine, cystine and cysteic acid, a doublet of doublets corresponding to the proton attached to the *α* carbon (H_α_ and H_Α_) and two doublets of doublets corresponding to the protons attached to the *β* carbon (H_β_ and H_Β_) can be observed [33, 34]. At the same pH, the chemical shifts of the cystine peaks (marked blue background) are slightly shifted downfield, i.e., at higher ppm, compared to those of cysteine, whereas the cysteic acid peaks are further shifted downfield compared to those of cystine. It is reasonable to assign the peaks observed after 3 min to cystine and other oxidized cysteine products. Over time, the peak intensity of cystine decreased, whereas the peak intensity of oxidized cysteine products gradually increased. In addition, a change of the peak H_A_ shape from a doublet‐like to a doublet of doublets was observed. This is probably due to the shift of the pH of the solution corresponds to the change of the oxidation state of the oxidized cysteine products from cysteine sulfenic acid to cysteic acid. The doublet‐like appearance observed at the beginning (most prominent at 3 min) is likely due to the averaging of peak shifts induced by pH changes over the course of the acquisition time. For the ^13^C NMR measurements, a highly concentrated aqueous solution containing 500 mm KCl, 20 mm HAuCl_4_, and 38 mm L‐cysteine was prepared as the detectable signal is very weak due to the low natural abundance (∼1.1%) of ^13^C nuclei, which can be observed by NMR [17]. Nevertheless, obtaining sufficient signals requires a long acquisition time and extensive signal integration, therefore, the results are considered to reflect the state reached after overnight reaction. As shown in Figure 2E, three main peaks in the ^13^C NMR spectra of the solution are observed at 36.1, 51.6, and 170.5 ppm, corresponding to the *β* carbon, the *α* carbon, and the carboxyl carbon, respectively. Comparison with the ^13^C NMR spectra of the cysteine, cystine and cysteic acid indicated that the carbon signals in the solution can be attributed to cystine and cysteic acid. This assignment is further supported by the slight downfield shift of the *β* carbon peak relative to that of cysteine, which is attributed to the increased electron‐withdrawing effect when the sulfur atom is bonded to another sulfur or oxygen atom, thereby shifting the *β*‐carbon peak to a higher chemical shift.

In order to analyze the interaction between [AuCl_4_]^−^ and cysteine, with a particular focus on the structure of the gold atom in the solution, the X‐ray absorption near edge structure (XANES) and extended X‐ray absorption fine structure (EXAFS) spectra of the electrolyte solution were measured. The XANES spectra at the Au L_3_‐edge are shown in Figure 2F. A prominent peak just after the absorption edge, known as the white line, is observed due to the electric dipole‐allowed electronic transition from the 2*p* orbital to the 5*d* orbital [35]. The position and intensity of the white line are directly related to the oxidation state of the Au atom and the density of unoccupied 5*d* states. The reaction begins with chloride exchange by cysteine and ultimately yields Au(I)‐like species. Consequently, the energy required to excite an electron from the orbital decreases, causing the white line to shift toward lower energy. Moreover, the reduction of the oxidation state decreases the density of unoccupied 5*d* orbitals, resulting in a reduction in the white line intensity. This result also supports the formation of Au(I)‐like species as a product of the disproportionation reaction. The Fourier transforms of the EXAFS spectra are shown in Figure 2G. The EXAFS spectra provide structural information about the local environment surrounding the absorbing atom, particularly the type, coordination number and bond distance of atoms in the first coordination shell. The peak position reflects the distance to the first nearest neighbors. The apparent first‐shell peak position shifted from ∼1.93 to ∼1.81 Å. This change reflects the alteration of the coordinating atoms of Au atom from Cl atom to S atom. Here, a peak appears at a very short distance (∼1.20 Å), which cannot be associated with any chemical bonding. This feature is well known in EXAFS spectra and arises from low‐frequency noise in the absorption data [36, 37]; therefore, the peak was disregarded. In addition, the peak intensity also changed, reflecting changes in the coordination number. These results are consistent with the ligand substitution between gold complexes and cysteine.

The use of reducing agents can also lead to the generation of Au(I)‐containing intermediates. Knowing that ascorbic acid itself is also chiral and its oxidation during the process for reduction of gold species introduces additional chemical species such as dehydroascorbic acid, potentially complicating the reaction system and interpretation of its chiral effects [38], we conducted the experiment in which Au(III)Cl_4_^−^ is pre‐reduced to Au(I)Cl_2_^−^ using ascorbic acid [39] before the addition of cysteine to verify the significance of the generation or presence of Au(I) species (Figure S7). As shown in Figure S7B, the resultant electrodeposited film exhibited a CD signal, indicating that chiral Au nanostructures can be formed even when Au(III) is predominantly pre‐reduced to Au(I) under the present conditions. This result supports the hypothesis that Au(I) chloride species such as Au(I)Cl_2_^−^ can act as precursors for cysteine‐mediated chiral growth.

In this study, we applied chiral Au nanostructures electrodeposited on Au corals as a platform for chiral sensing. To evaluate their surface‐enhanced Raman scattering (SERS) performance, we examined four samples: (i) a Si substrate, (ii) Au corals electrodeposited on an ITO‐ coated glass substrate, (iii) chiral Au nanostructures electrodeposited on an ITO‐coated glass substrate, and (iv) chiral Au nanostructures electrodeposited on Au corals/ITO‐coated glass substrate. In this study, the samples were immersed in the analyte solution, and a focused laser beam with a wavelength of 785 nm was irradiated onto the sample using an objective lens. The resulting SERS signals were collected through the same objective lens as illustrated in Figure 3A. A blank SERS spectrum of the l‐chiral Au/coral Au/ITO substrate measured in DIW is shown in Figure S8. No distinct peaks attributable to residual species remaining on the substrate after rinsing were observed. We measured the SERS signals from an analyte solution of 1 mm rhodamine 6G dissolved in ethanol. As shown in Figure 3B, the Au corals clearly enhance the SERS signals. Furthermore, the SERS signals from the chiral Au nanostructures and the chiral Au nanostructures on Au corals were 5.09 and 53.6 times more intense, respectively, than those from the Au corals alone. (Here, the enhancement performance was evaluated using the Raman peak at 1509 cm^−1^, which is attributed to C─C stretching vibration of the aromatic ring. The assignments of the molecular vibrations corresponding to each Raman peak are summarized in Table S1 [40, 41].) It is noteworthy that the SERS signals from the chiral Au nanostructures formed on the Au corals are enhanced beyond the sum of the individual enhancements provided by the Au corals and the chiral Au nanostructures. We initially hypothesized that the difference in the enhancement could be simply due to the difference in the surface area of the nanostructures in each film. To evaluate this, we estimated the effective surface area of each film electrochemically by performing cyclic voltammetry (CV) measurements in an acidic medium (0.5 m H_2_SO_4_) as shown in Figure S9. The cathodic peak around 0.9 V corresponds to the reduction of Au oxide species and was used to calculate the surface area. The relative surface areas of the chiral Au nanostructures and the chiral Au nanostructures on Au corals were found to be 3.07 and 3.69 times larger, respectively, than that of the Au corals. However, these values do not correlate with the observed SERS enhancement. This indicates that the pronounced SERS enhancement is not solely due to surface area, but rather to a synergistic effect between the Au corals and the chiral Au nanostructures. A cross‐sectional transmission electron microscopy (TEM) image of the chiral Au nanostructures on the Au corals is shown in Figure S10. The chiral Au nanostructures exhibit complex dendritic morphologies rather than well‐defined helical geometries. Moreover, their substantial overlap made it difficult to unambiguously reconstruct and determine their three‐dimensional morphology using electron tomography. Therefore, conventional geometrical parameters for regular helical structures, such as the pitch and helix angle, cannot be rigorously defined for the present system. Nevertheless, the cross‐sectional TEM image revealed a characteristic spacing of approximately 20 nm between the constituent structural units. In addition, a small gap of several nanometers was observed between the chiral Au nanostructures and the Au corals. This gap may contribute to the synergistic SERS enhancement.

**FIGURE 3:**
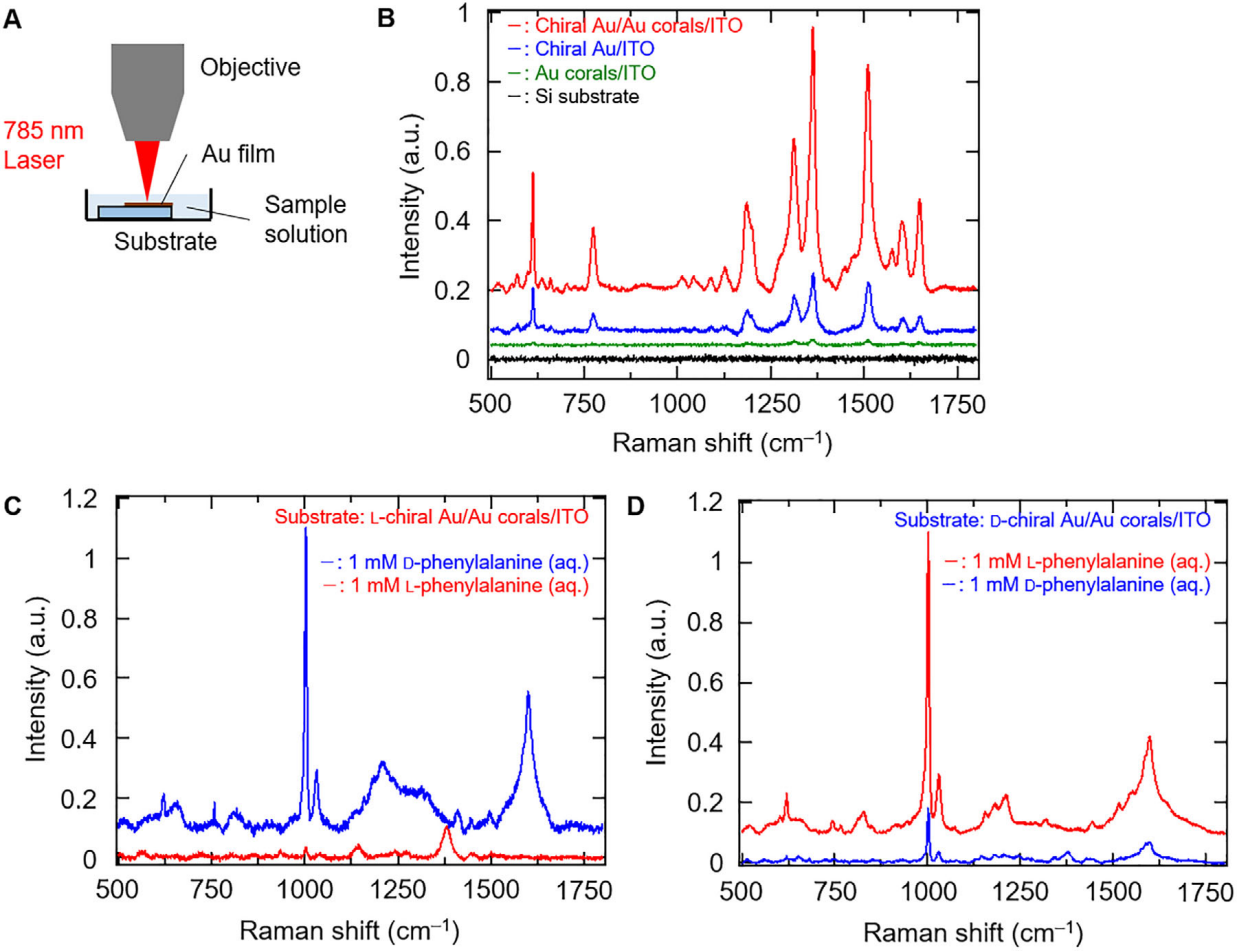
(A) Schematic image of the SERS measurements in this study. The samples were immersed in the analyte solution, and a focused laser beam with a wavelength of 785 nm was irradiated onto the sample using an objective lens. The resulting SERS signals were collected through the same objective lens. (B) SERS spectra of 1 mm rhodamine 6G dissolved in ethanol obtained from each substrate. (C,D) SERS spectra of 1 mm L‐phenylalanine (aq.) (red) and D‐phenylalanine (aq.) (blue) measured on chiral gold nanostructures synthesized using L‐ (C) and D‐cysteine (D) on the Au corals. Chiral gold nanostructures synthesized using L‐ and D‐cysteine are referred to as “L‐chiral Au” and “D‐chiral Au”, respectively.

SEM evidence of complex dendritic morphology and CD evidence of the chiroptical response and the strong SERS enhancement of the chiral Au nanostructures on the Au corals prompted us to explore their potential chiroptical functionalities, specifically the so‐called SERS‐chiral anisotropy (SERS‐ChA) effects [42, 43]. Therefore, we examined the chiral discrimination capability of the films. We measured the SERS signals using an analyte solution of 1 mM phenylalanine dissolved in DIW, as illustrated in Figure 3A. Enantiomer‐dependent differences in SERS intensity depending on the L‐ and D‐phenylalanine enantiomers were observed for both chiral Au nanostructures synthesized using L‐ and D‐cysteine on the Au corals. These results suggest that the interactions between the analyte enantiomers and the chiral Au nanostructures are enantioselective and exhibit distinct chiral recognition behavior. Although Raman peaks originating from various molecular vibrations were observed (the assignments of the peaks are summarized in Table S2) [44, 45, 46], both the peak positions and intensities varied between measurements, even within the same region of the same sample. Therefore, we focused on the peak at 1002 cm^−1^, which is attributed to the phenyl‐ring‐breathing mode and shows relatively high reproducibility. Due to its prominence and consistency, this peak was considered suitable for chiral discrimination. This high reproducibility is probably due to the stability and rigidity of the phenyl ring of phenylalanine, which arises from the delocalization of π‐electrons within its aromatic ring structure. Furthermore, the phenyl‐ring‐breathing mode of the phenyl ring possesses a large Raman scattering cross‐section, making it consistently and strongly observed in SERS measurements, and thereby more prominent and reproducible than other vibrational modes [47].

It is important to note that the enantiomer that gives the larger response generally depends on the analyte molecule. The l/d notation or *R*/*S* notation for a molecule and that for a nanostructure just represent the stereochemical configuration or twisting direction within their respective systems. In this study, the l/d notation for the Au substrate is based on the chirality of cysteine employed, simply to distinguish between the two chiral structures, but it should be noted here that this system is not an example of molecular imprinting. Therefore, the use of the same designation does not necessarily imply geometrical or chemical complementarity. Consequently, whether a same‐handed or opposite‐handed combination produces a stronger response varies depending on the analyte.

We also measured the CD spectra of l‐ and d‐phenylalanine adsorbed on both l‐ and d‐chiral Au/coral Au/ITO substrates (Figure S11). Consistent with the SERS measurements, d‐phenylalanine exhibited a stronger CD response on the l‐chiral Au/coral Au/ITO substrate, whereas l‐phenylalanine exhibited a stronger CD response on the d‐chiral Au/coral Au/ITO substrate. These results also indicate that the enantiomer exhibiting the stronger CD response is determined by the specific interaction between the analyte and the chiral Au surface and is not necessarily the enantiomer with the same l/d designation as the substrate.

The advantage of our chiral Au nanostructures on the Au corals lies in their “*intrinsic*” chiral nature of the Au nanostructures. This enables the expression of chiral discrimination ability without the need for any post‐treatment such as molecular imprinting by the chiral molecules [48] to be identified, chirality transfer of supramolecular structures, or surface modification of the gold nanostructures with self‐assembled monolayers (SAMs) [49, 50] or metal–organic frameworks (MOFs) [14]. Therefore, elucidating which types of chiral molecules can be discriminated using this structure presents a highly intriguing question. To investigate the applicability of this substrate to other molecules in their chiral discrimination, we additionally conducted SERS measurements for 1 mm aqueous solutions of tryptophan, tyrosine, valine, and serine, which possess 3‐indolylmethyl (‐CH_2_C_8_H_6_N), *para*‐hydroxyphenylmethyl (‐CH_2_C_6_H_4_OH), isopropyl (‐CH(CH_3_)_2_), and hydroxymethyl (‐CH_2_OH) groups, respectively. These amino acids were selected as representative examples of molecules with relatively hydrophobic‐aromatic, polar‐aromatic, hydrophobic‐nonaromatic and polar‐nonaromatic, respectively [51]. As shown in Figure S12, clear enantioselective differences were observed for tryptophan and tyrosine, both of which contain aromatic groups, whereas valine and serine, which do not contain aromatic groups, exhibited no pronounced differences between the enantiomers. Because the SERS intensity depends not only on the number of adsorbed molecules and their distance from the Au surface but also on their adsorption orientation and coupling with the local electromagnetic field, the observed enantioselectivity cannot be attributed solely to the strength of the adsorption interaction. Therefore, it may be difficult to simply and universally explain regarding the molecular structures for chiral discrimination. Nevertheless, together with the results obtained for phenylalanine, these findings indicate that the chiral Au substrate developed in this study can discriminate between the enantiomers of the aromatic amino acids under the present experimental conditions. Since the discrimination capability of these chiral substrates has been verified for several types of amino acids in this study, systematic measurements of a wider variety of chiral compounds will be required to comprehensively identify the chemical and structural features that enable enantiomeric discrimination using this substrate.

## 4 Conclusions

In this study, we developed a facile electrochemical strategy to construct hierarchical plasmonic architectures, in which chiral Au nanostructures were integrated onto Au coral frameworks directly grown on ITO‐coated glass substrates. By controlling the substitution of chloride ions in [AuCl_4_]^−^ with cysteine in the electrolyte solution and the subsequent reduction of Au(III) complexes, we achieved precise morphological control of the resulting Au nanostructures, which exhibited CD. Furthermore, SERS measurements on these chiral Au nanostructures demonstrated their potential as substrates for chiral discrimination. Overall, these results underscore the versatility of electrochemical synthesis for constructing chiral plasmonic architectures, advancing the fundamental understanding of electrochemical chiral growth while simultaneously opening new opportunities for practical chiral sensing devices, enantioselective catalysis, and next‐generation optoelectronic applications.

## Author Contributions

**Junhe Wang**: writing – review and editing, methodology, data curation, investigation, validation, formal analysis, visualization. **Yuta Shiga**: methodology, data curation, investigation, validation, formal analysis, visualization. **Takuya Nakanishi**: writing – review and editing, formal analysis, visualization, methodology, validation, supervision, conceptualization. **Tianji Zhang**: methodology, data curation, investigation, validation, formal analysis, visualization. **Olga Guselnikova**: methodology. **Toru Asahi**: resources, project administration, funding acquisition, supervision, validation, writing – review and editing. **Yusuke Yamauchi**: writing – review and editing, supervision, resources, project administration, funding acquisition, conceptualization, methodology, validation, formal analysis, visualization. **Kenta Nakagawa**: writing – review and editing, supervision, resources, project administration, funding acquisition, conceptualization, methodology, validation, formal analysis, visualization.

## Conflicts of Interest

The authors declare no conflicts of interest.

## Supporting information




**Supporting File**: smll75471‐sup‐0001‐SuppMat.pdf.

## Data Availability

The data that support the findings of this study are available from the corresponding authors upon reasonable request.
