# Supplementary material for: Synthesis of Chiral Gold Nanostructures on Gold‐Coral‐Coated Transparent Conductive Oxide Substrates via [AuCl4]– and Cysteine Interactions for Enhanced Chiral Sensing
Source: Small. 2026 Sep 3;22(61):e75471. doi: 10.1002/smll.75471 (PMC13725400; doi:10.1002/smll.75471)
Supplement: Supplementary file 1 [file SMLL-22-e75471-s001.pdf]

Supporting Information

Synthesis of Chiral Gold Nanostructures on Gold-Coral-Coated Transparent Conductive Oxide Substrates via $[AuCl_4]^-$ and Cysteine Interactions for Enhanced Chiral Sensing

Kenta Nakagawa,[1,*] Junhe Wang,[2] Tianji Zhang,[2] Yuta Shiga,[2] Olga Guselnikova,[3] Takuya Nakanishi,[1] Yusuke Yamauchi,[3,4,5,*] Toru Asahi[1,2,6*]

[1]Comprehensive Research Organization, Waseda University, 2-2 Wakamatsu-cho, Shinjuku-ku, Tokyo 162-8480, Japan.

[2]Faculty of Science and Engineering, Waseda University, 2-2 Wakamatsu-cho, Shinjuku-ku, Tokyo 162-8480, Japan.

[3]International Center for Materials Nanoarchitectonics (WPI-MANA), National Institute for Materials Science (NIMS), 1-1 Namiki, Tsukuba, Ibaraki, 305-0044, Japan.

[4]Department of Materials Science and Engineering, School of Engineering, Nagoya University, Furo-cho, Chikusa-ku, Nagoya 464-8601, Japan.

[5]Australian Institute for Bioengineering and Nanotechnology (AIBN), The University of Queensland (UQ), Brisbane, QLD 4072, Australia.

[6]Research Organization for Nano & Life Innovation, Waseda University, 513 Wasedatsurumaki-cho, Shinjuku-ku, Tokyo 162-0041, Japan.

*Corresponding authors: kenta.nakagawa@aoni.waseda.jp, y.yamauchi@nagoya-u.jp, tasahi@waseda.jp

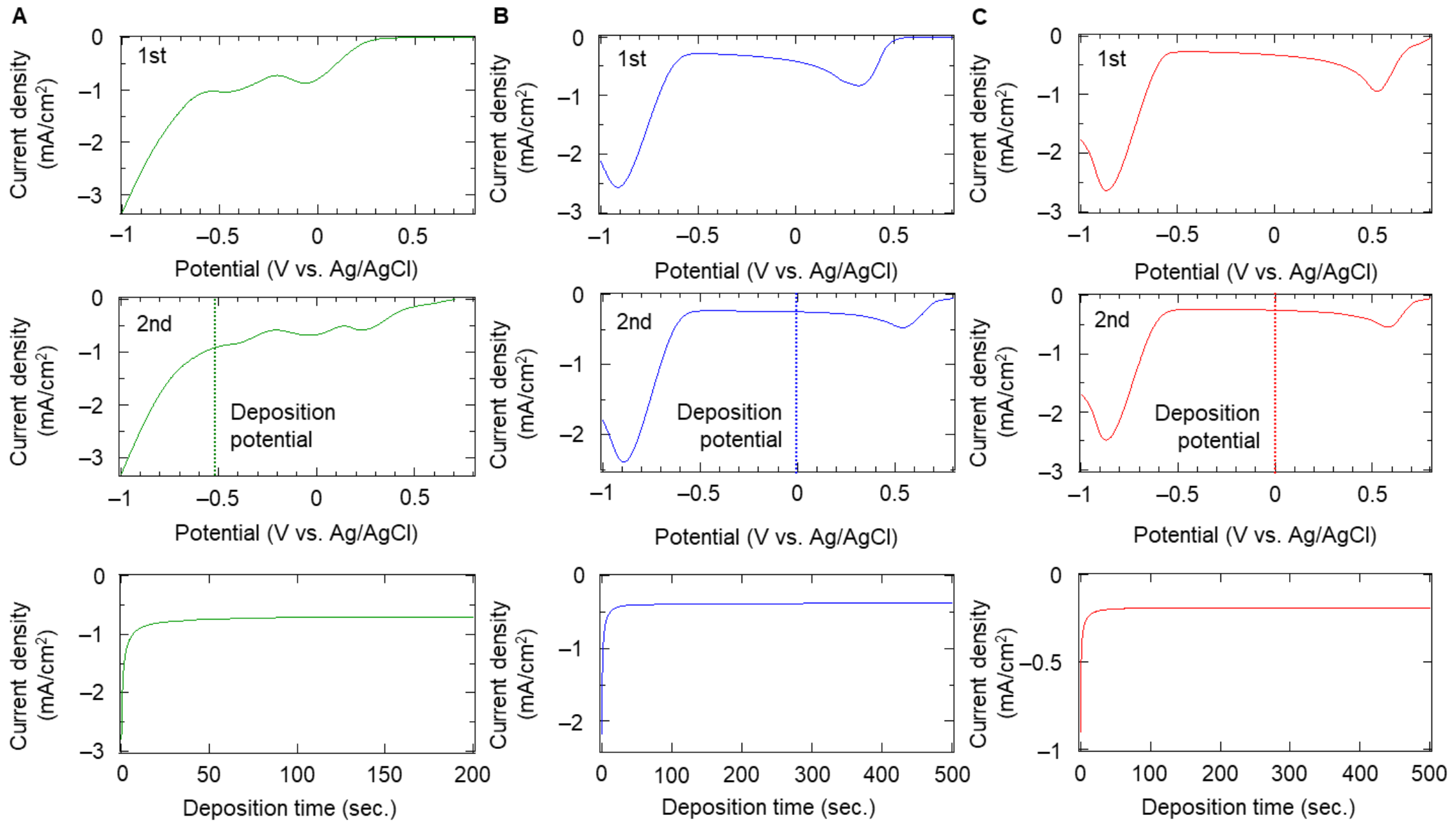

Figure S1. Linear sweep voltammetry (LSV) plots (top and middle) and amperometric curves (bottom) for the electrodeposition of Au corals on ITO-coated glass substrate (A), chiral Au nanostructures on ITO-coated glass substrate (B), and chiral Au nanostructures on Au corals (C), respectively. For each deposition, LSV was performed twice, and the deposition potential was determined based on the second LSV curve.

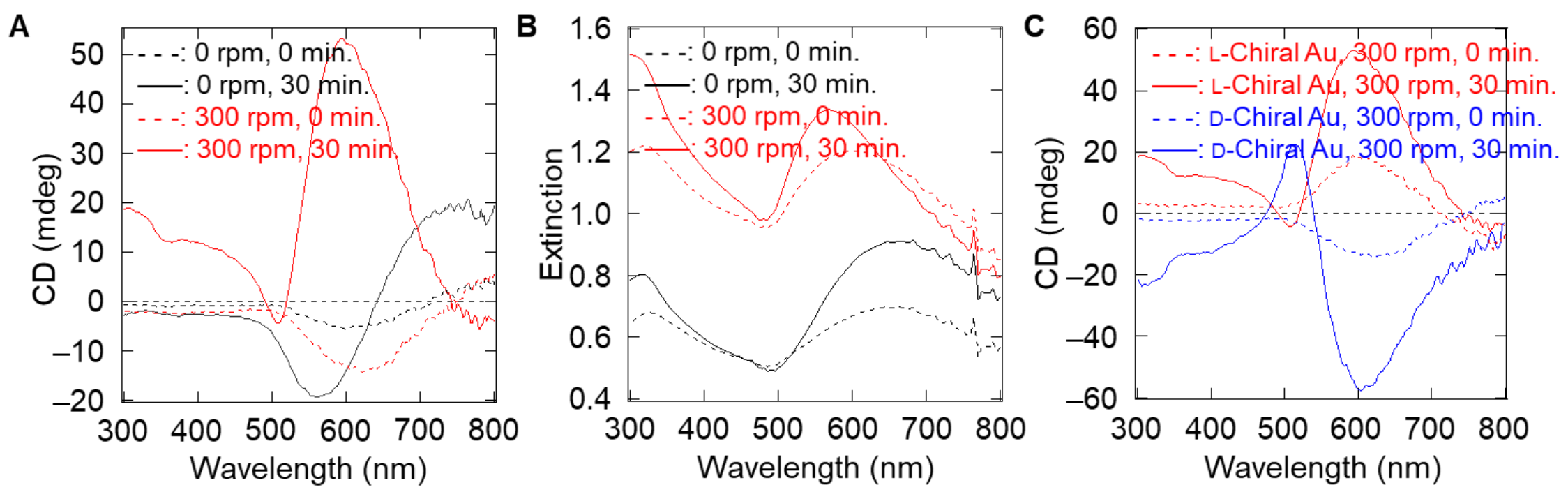

Figure S2. CD spectra (A,C) and UV-Vis absorption spectra (B) of chiral Au nanostructures electrodeposited on ITO-coated glass substrates under stirred (300 rpm) and unstirred (0 rpm) conditions, with and without allowing the electrolyte solution to stand for 30 min before electrodeposition. The black and red curves represent chiral gold nanostructures synthesized using L-cysteine, whereas the blue curve represents chiral gold nanostructures synthesized using D-cysteine. Samples synthesized with stirring also exhibited large CD signals. However, even under stirred conditions, the interactions between the Au species and cysteine that develop while the electrolyte solution is allowed to stand substantially affect the chiroptical response of the resulting films.

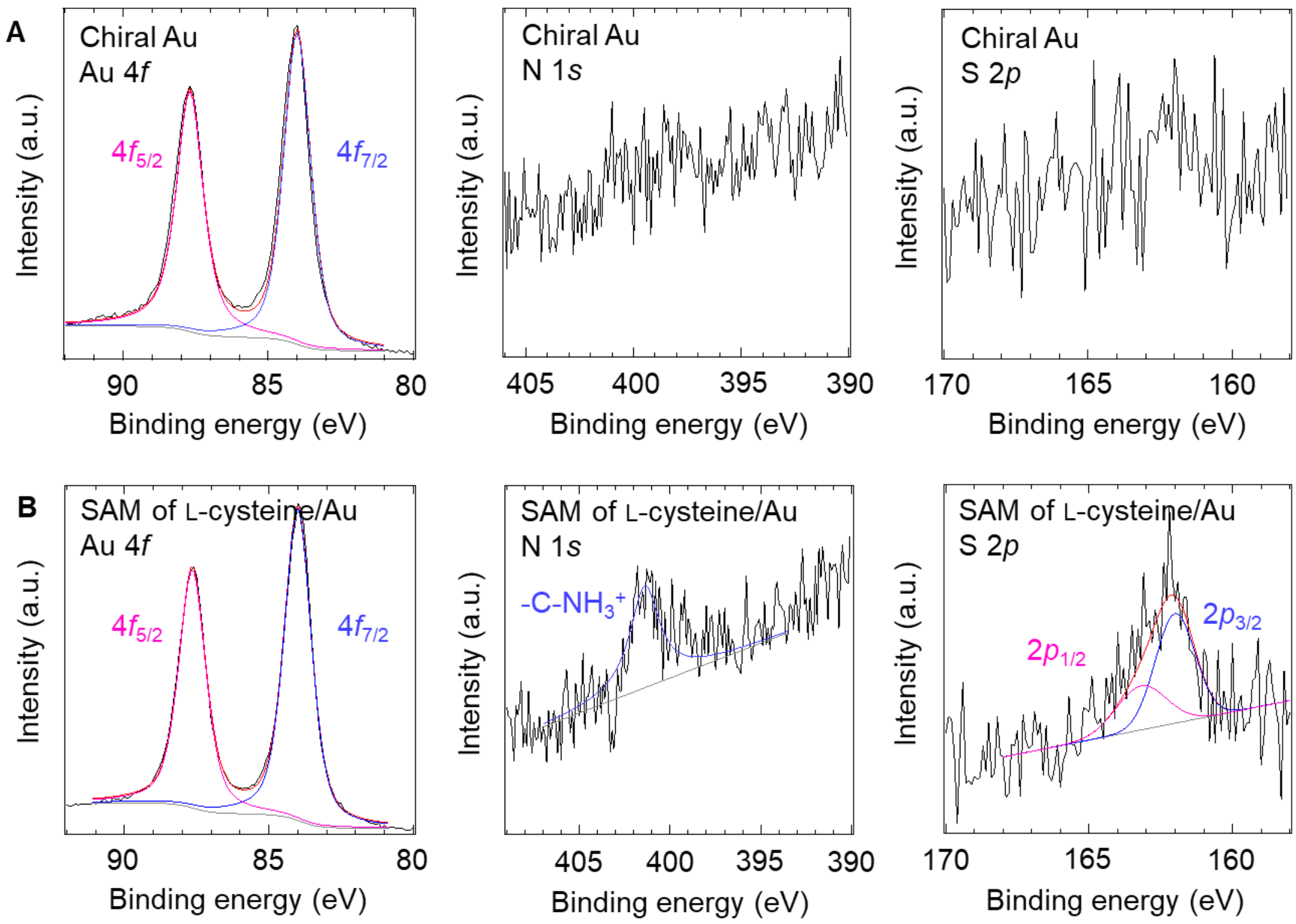

Figure S3. X-ray photoelectron spectroscopy (XPS) spectra of the chiral Au/ITO substrate (A) and a self-assembled monolayer of L-cysteine on Au. The black, red, and gray curves represent the experimental spectra, the overall fitted spectra, and the backgrounds, respectively. Shirley's method was utilized to subtract the background. During the fitting, the Au $4f_{5/2}$ to $4f_{7/2}$ peak-area ratio was constrained to 3:4, whereas the S $2p_{1/2}$ to $2p_{3/2}$ peak-area ratio was constrained to 1:2.

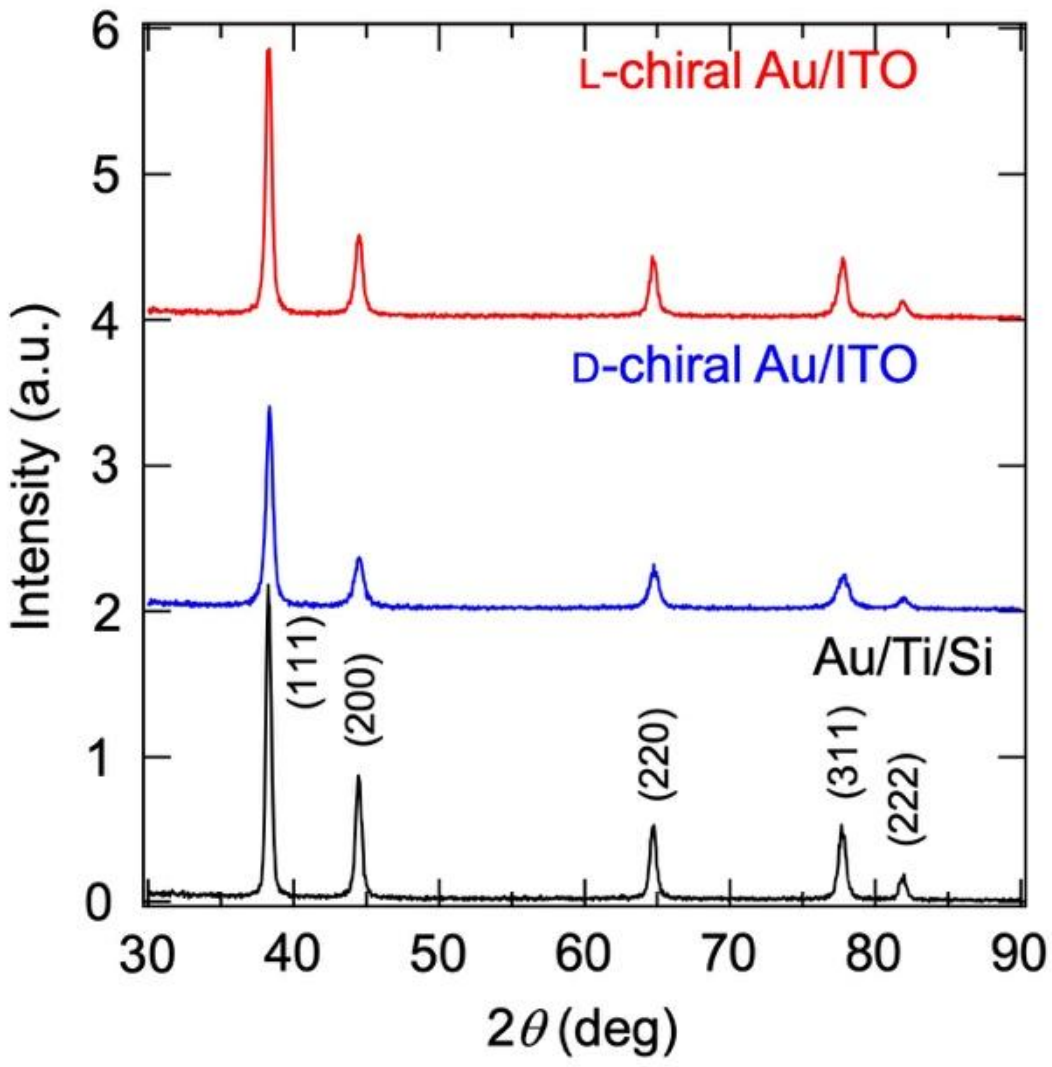

Figure S4. XRD patterns of Au/Ti-deposited Si substrate (black) and chiral Au nanostructures synthesized on ITO-coated glass substrates using L-cysteine (red) and D-cysteine (blue).

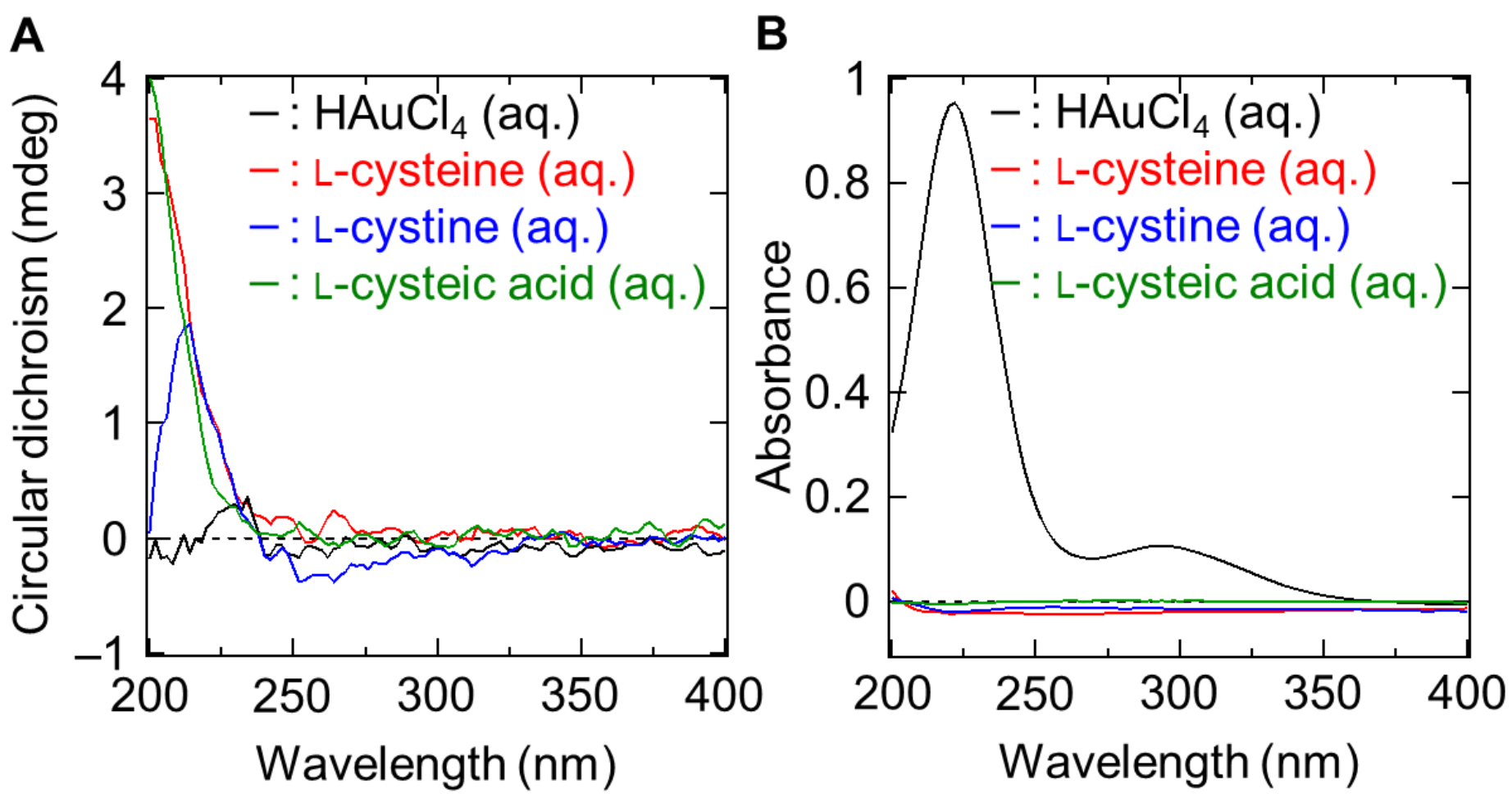

Figure S5. Circular dichroism (A) and UV absorption (B) spectra of 0.4 mM $HAuCl_4$ (aq.) (black) and 0.4 mM L-cysteine (aq.) (red), L-cystine (aq.) (blue) and L-cysteic acid (aq.) (green).

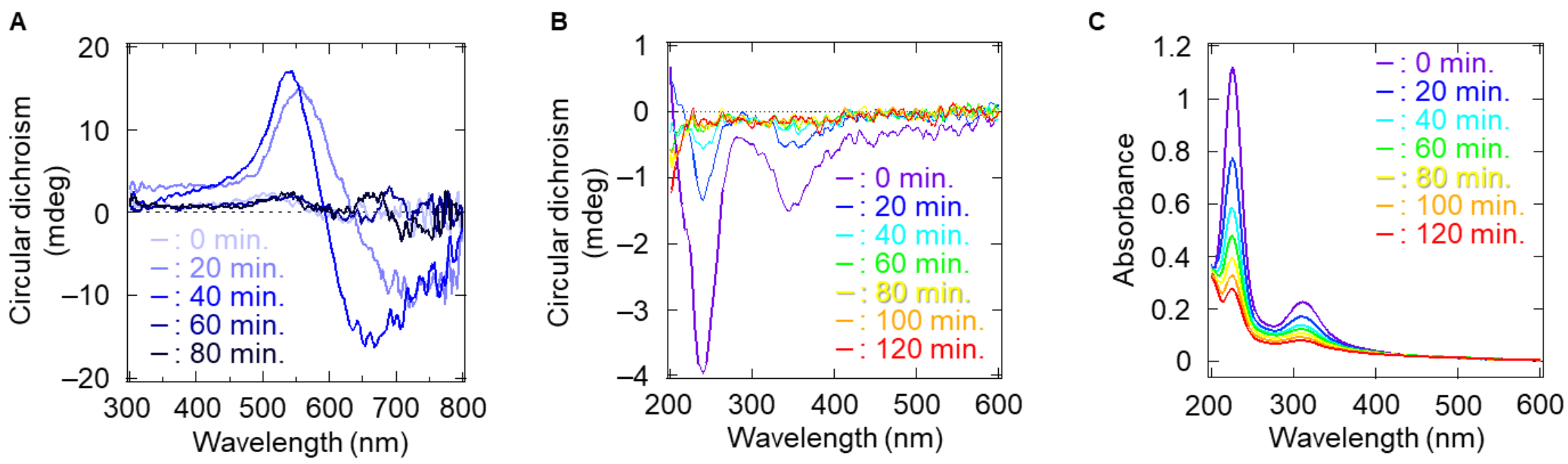

Figure S6. Time-dependent spectroscopic analyses of the interaction between $[AuCl_4]^-$ and cysteine. (A) CD spectra of chiral Au nanostructures synthesized on Au corals as a function of the waiting time between the mixing of aqueous KCl, $HAuCl_4$, and D-cysteine solutions and the start of electrodeposition. (B,C) Time-dependent CD (B) and UV-Vis (C) spectra of a mixed aqueous solution containing 15 mM KCl, 0.6 mM $HAuCl_4$, and 0.3 mM D-cysteine at room temperature.

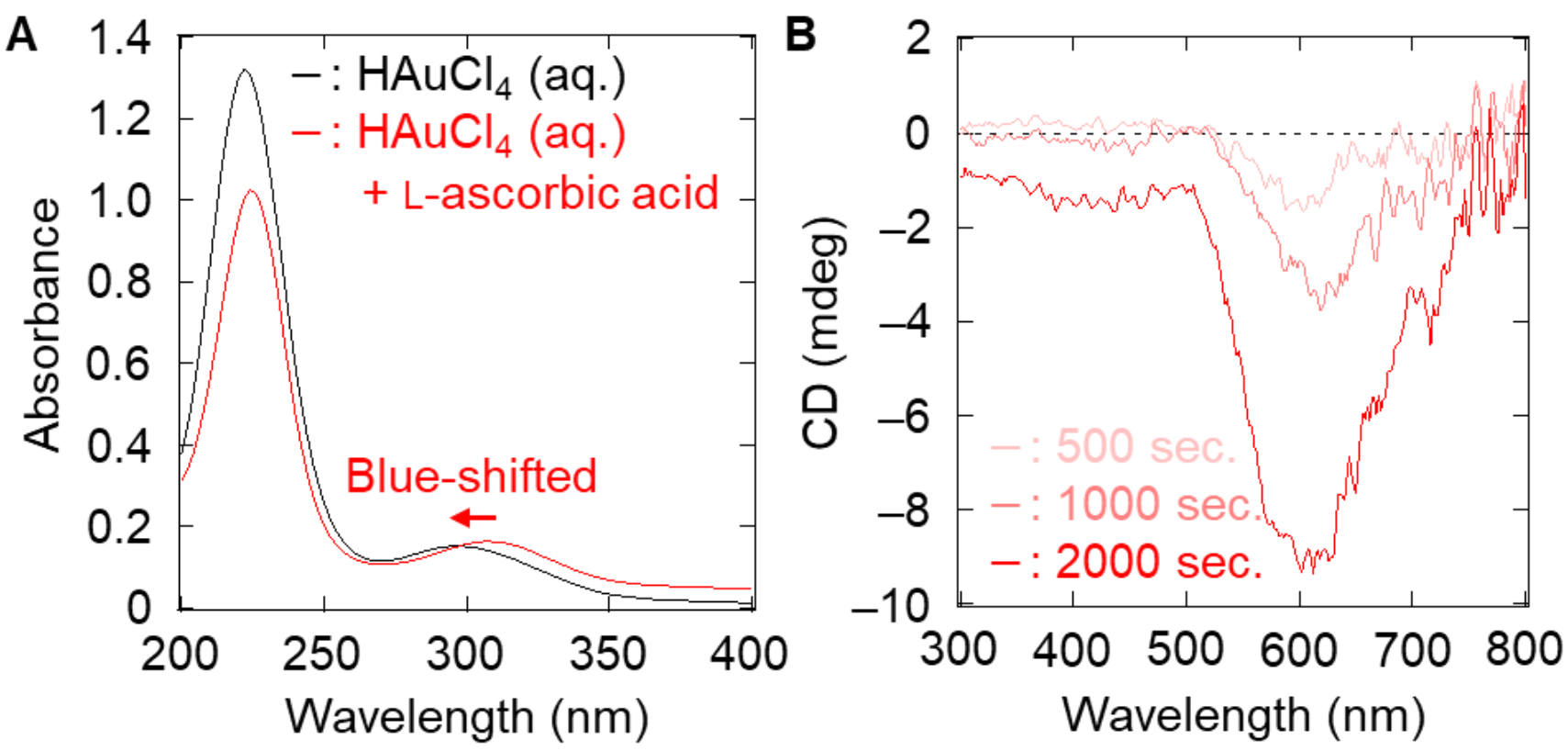

Figure S7. (A) UV-Vis spectra of a 0.5 mM $HAuCl_4$ (aq.) solution (black) and the same solution after the addition of 0.88 mg of L-ascorbic acid (red). The color of the solution changed from yellow to colorless, accompanied by the disappearance of the characteristic visible absorption of $AuCl_4^-$. No precipitation was observed, suggesting that $AuCl_4^-$ containing Au(III) was predominantly reduced to $AuCl_2^-$ containing Au(I), rather than to metallic Au(0).[39] (B) CD spectrum of the electrodeposited film obtained from an electrolyte solution containing 0.1 M KCl (aq.), 2 mM L-ascorbic acid (30 mg)-treated $HAuCl_4$ (aq.), and 0.4 mM L-cysteine (aq.). Electrodeposition was initiated immediately after preparation of the electrolyte solution and continued for several hundred seconds. The resulting electrodeposited film exhibited a CD signal, indicating that chiral Au nanostructures can be formed even when the Au(III) precursor is predominantly pre-reduced to Au(I) under the present conditions.

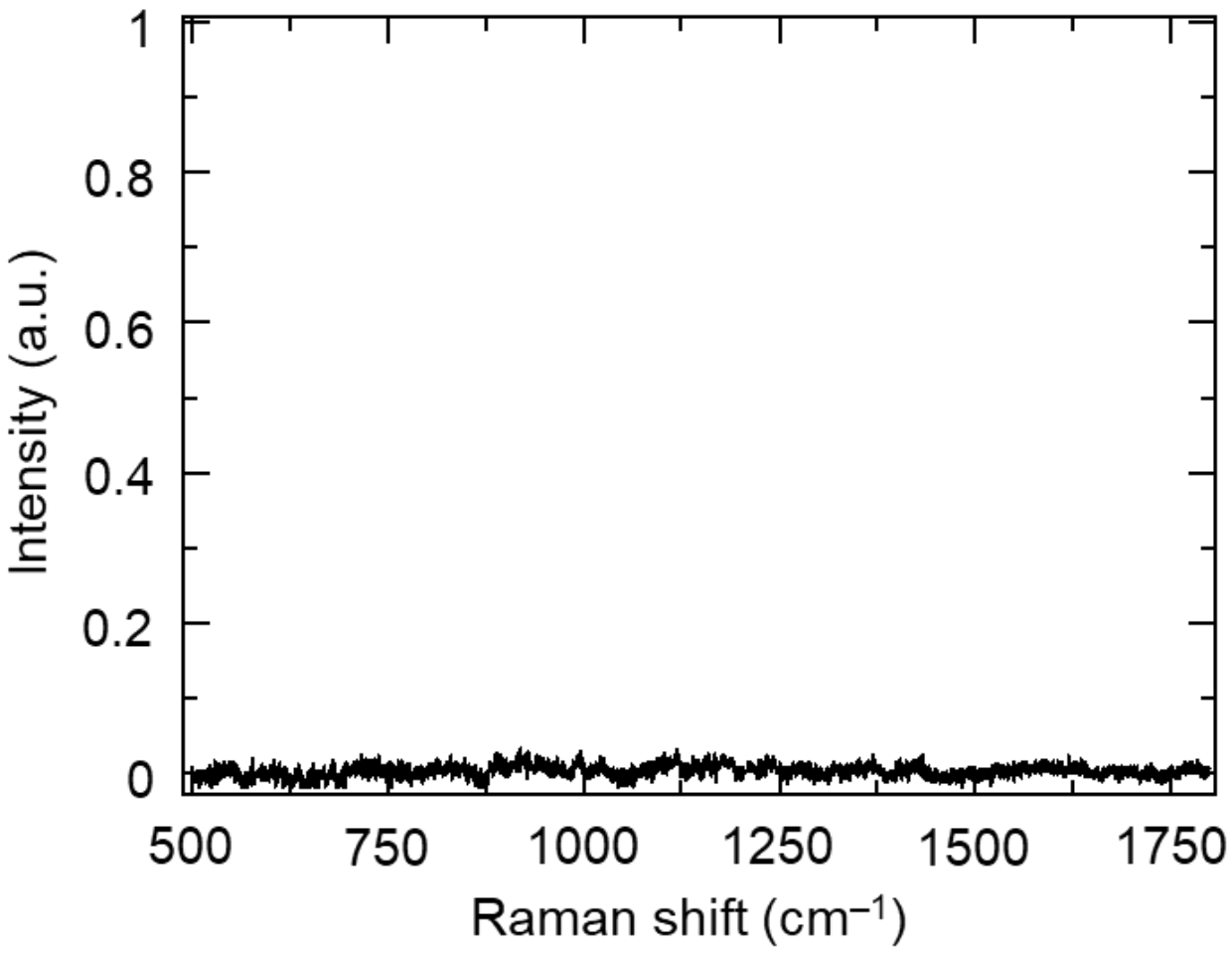

Figure S8. Blank SERS spectrum of the L-chiral Au/coral Au/ITO substrate measured in DIW. No distinct peaks attributable to residual species remaining on the substrate after rinsing were observed.

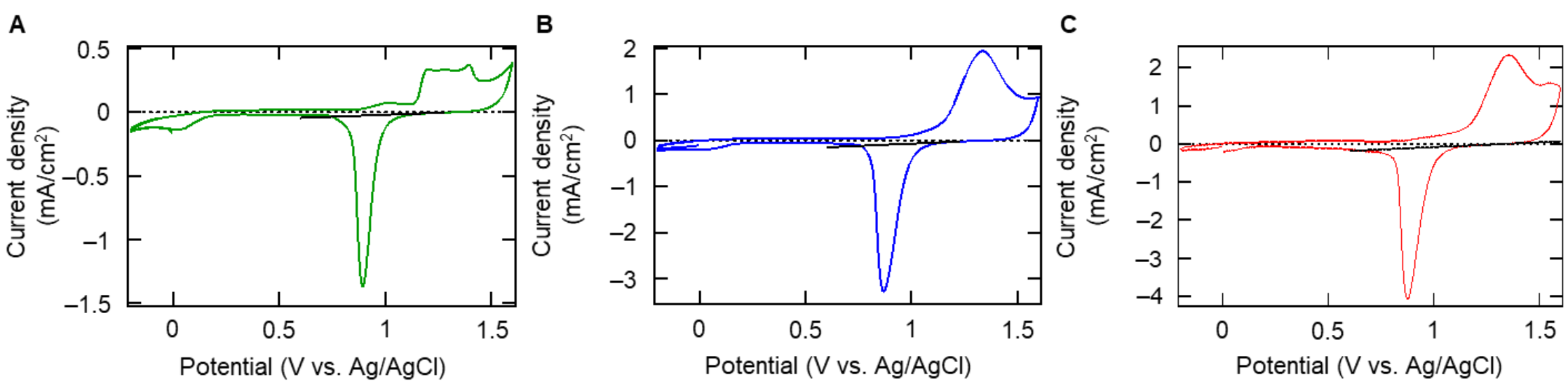

Figure S9. Cyclic voltammetry (CV) plots for Au corals on ITO-coated glass substrate (A), chiral Au nanostructures on ITO-coated glass substrate (B), and chiral Au nanostructures on Au corals (C), measured in an acidic medium (0.5 M H_2SO_4). The cathodic peak around 0.9 V corresponds to the reduction of Au oxide species and was used to calculate the surface area. The relative surface areas of the chiral Au nanostructures and the chiral Au nanostructures on Au corals were found to be 3.07 and 3.69 times larger, respectively, than that of the Au corals.

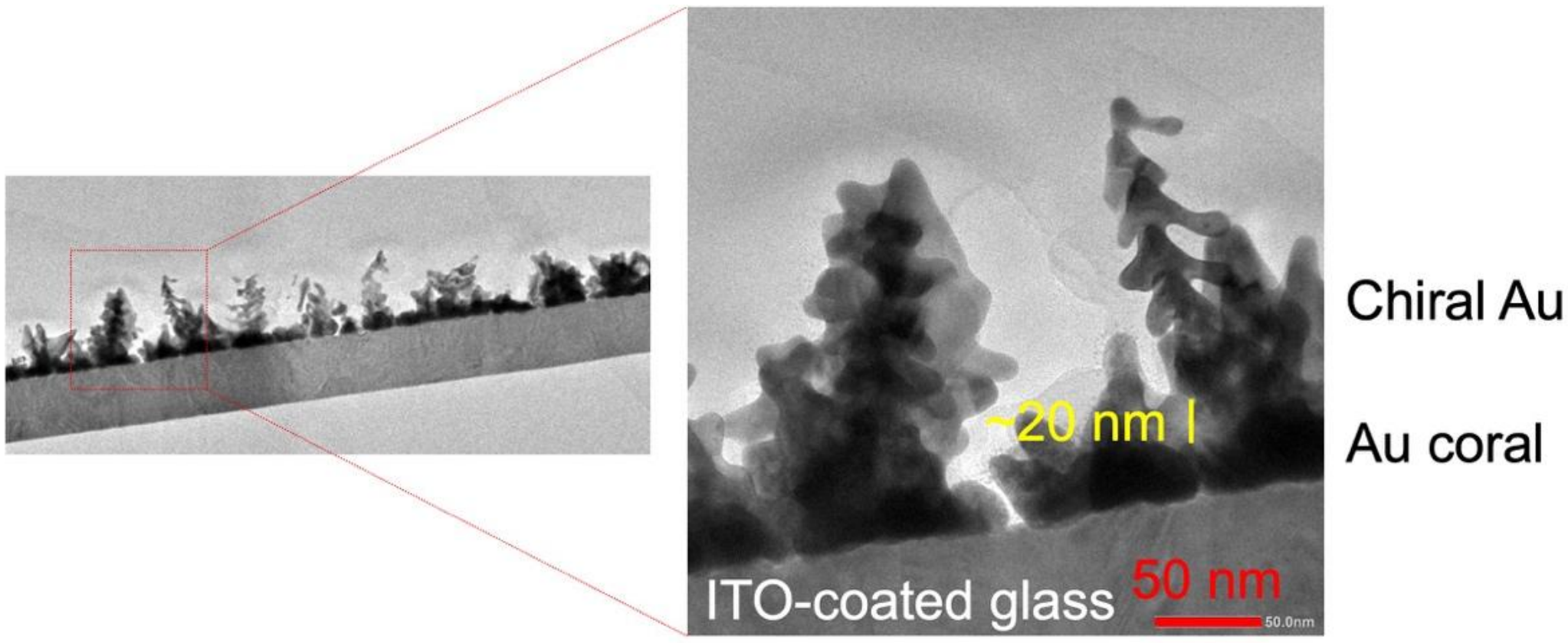

Figure S10. Cross-sectional transmission electron microscope (TEM) images of chiral Au nanostructures synthesized on Au corals.

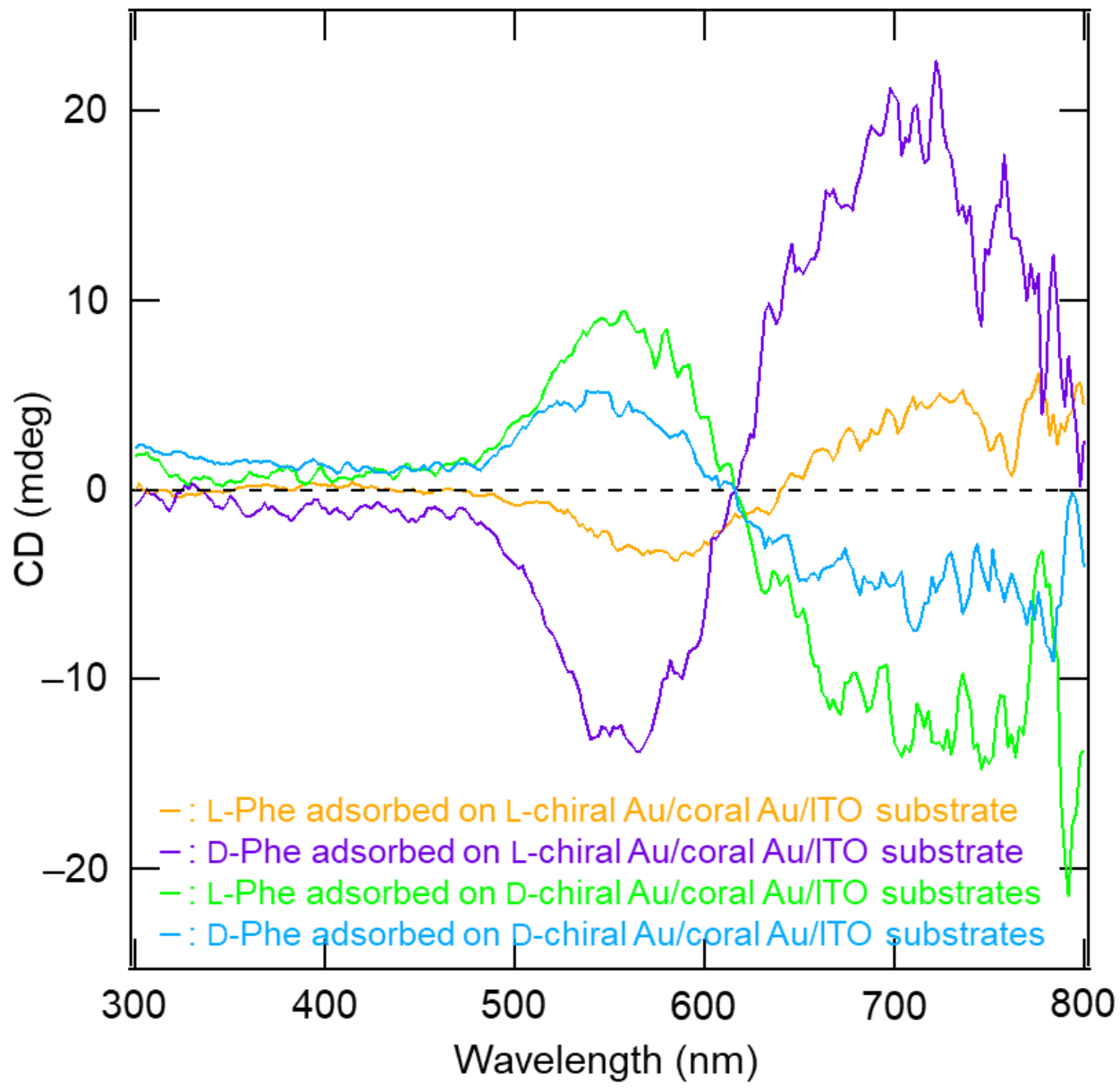

Figure S11. CD spectra of L- and D-phenylalanine adsorbed on both L- and D-chiral Au/coral Au/ITO substrates.

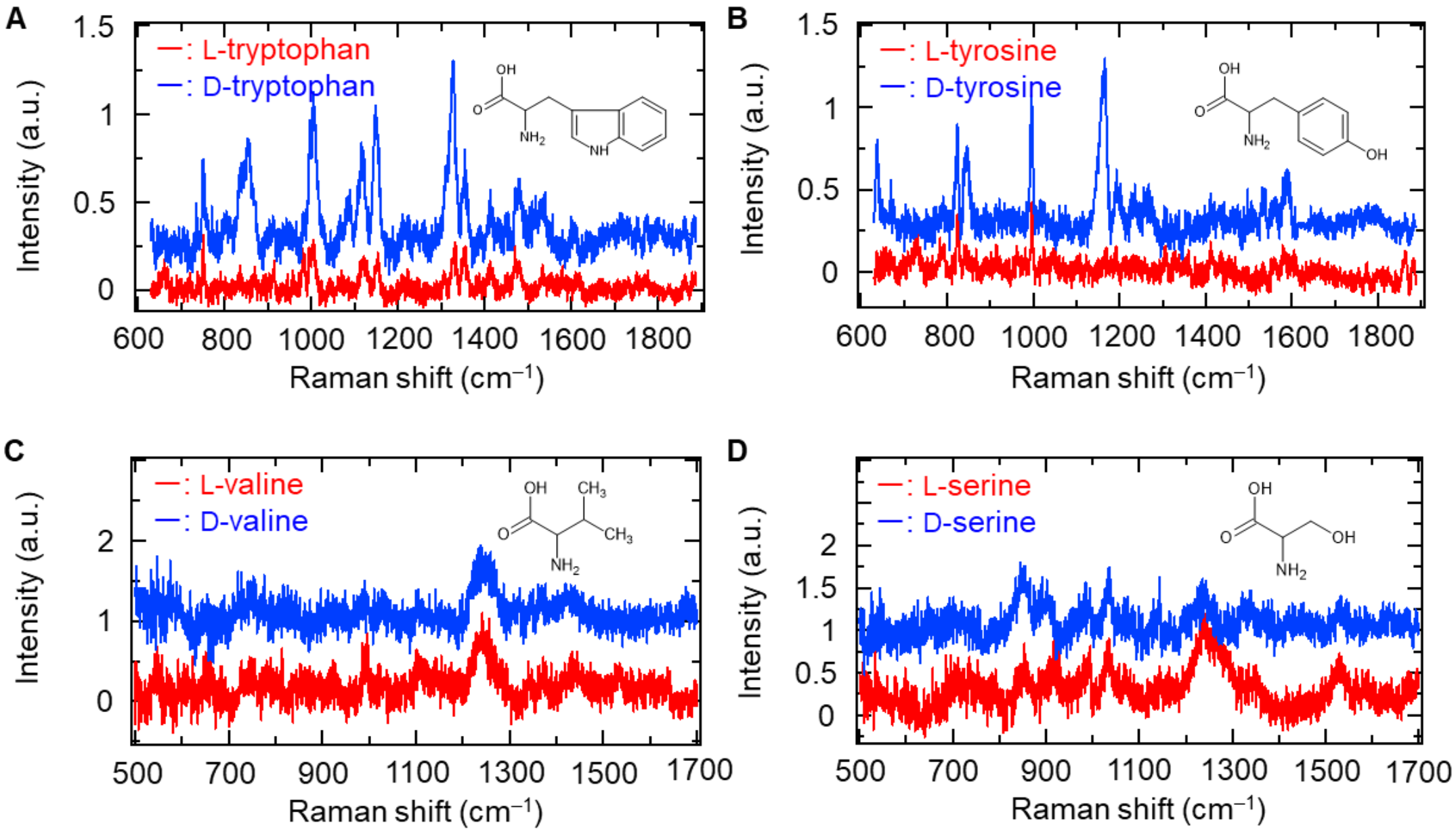

Figure S12. SERS spectra of 1 mM L/D-tryptophan (aq.)(A), L/D-tyrosine (aq.)(B), L/D-valine(aq.)(C), L/D-serine (aq.)(D) obtained from chiral gold nanostructures synthesized using L-cysteine on the Au corals. The spectra are vertically offset for clarity.

Table S1. Comparison of the R6G SERS bands observed in this study with reported Raman and SERS bands and their vibrational assignments.[40,41]

In this study	Raman (cm^{-1})	SERS (cm^{-1})	Assignment
611	613	611	ν(XRD) in-plane/out-of-plane
774	775	775	δ(C–H) out-of-plane, ν(XRD) in-plane
1003	1013	1011	External group mode (EGM)
1187	1184	1178	ν(XRD) in-plane, δ(C–H), δ(N–H)
1311	1312	1310	ν(XRB) in-plane, δ(N–H), ω(CH_2)
1362	1364	1361	ν(XRS, C–C), δ(C–H) in-plane
1509	1512	1505	ν(XRS), ν(C–N), δ(C–H), δ(N–H)
1572	1577	1577	ν(XRS), δ(N–H) in-plane
1600	1590	1598	ν(XRS, C–C)
1647	1651	1649	ν(XRS), δ(C–H) in-plane

Note: ν: stretching vibration, δ: bending vibration, ω: wagging vibration, XRD: xanthene ring deformation, XRB: xanthene ring breathing, XRS: xanthene ring stretching

Table S2. Comparison of the phenylalanine SERS bands observed in this study with reported Raman and SERS bands and their vibrational assignments.[44-46]

In this study	Raman (cm^{-1})	SERS (cm^{-1})	Assignment
620	622		Phenyl ring breathing
	751		Phenyl ring breathing
		762	δ_b(COOH)
	771		Out-of-plane C-H bending of the phenyl ring
817	817		C_β-centered side-chain angular deformation
817		822	ν(C-C), $\tau(CH_2)$, and/or $\rho(NH_3^+)$
		856	ν(C–C), $\tau(CH_2)$, and/or $\rho(NH_3^+)$
	858		$\nu(C_\alpha–C_t)$, $\nu(N_t-C_\alpha)$, or their coupling
926	922		$\nu(C_\alpha–C_t)$, $\nu(N_t-C_\alpha)$, or their coupling
926		917	out-of-plane C-H bending of the phenyl ring (Phe ν_5)
1002	1004		Phenyl ring breathing (ν_{12})
1002		998	Phenyl ring breathing (ν_{12})
1031	1032		$\nu(C_\varepsilon-C_\zeta)$ in-phase coupled with $\delta(C_\zeta-C_\varepsilon$-H) in-phase
1031		1025	Phenyl-ring C-C stretching coupled with in-plane C-H bending (ν_{18a})
	1068		$\nu(C_\alpha–C_t)$, $\nu(N_t-C_\alpha)$, or their coupling
		1074	$\tau(NH_3^+)$ and/or $\omega(CH_2)$
1145?		1130	$\omega(NH_3^+)$
1145?, 1160	1160		δ(C–H)
1209	1207	1204	$\nu(C_\beta–C_\gamma)$ coupled with in-phase phenyl-ring C-C stretching (Phe ν_{7a})
	1263		$\delta(C_\beta–C_\alpha$–H), $\tau(C_\beta H)$, $\rho(C_\beta H)$, $\nu_s(COO^-)$
		1306	$\omega(C\alpha_2H_2)$
	1327		C_β-centered side-chain angular deformation
	1360		$\delta(N–C_\alpha$–H), $\delta(C_\beta–C_\alpha$–H), $\rho(C_\beta H)$, $\tau(C_\beta H)$

Note: ν: stretching vibration, δ: bending vibration, ω: wagging vibration, τ: twisting vibration; and ρ: rocking vibration. C_t and N_t denote the carbon and nitrogen atoms, respectively, in the terminal charged groups of the phenylalanine backbone.
